# An Acute Bout of Quadriceps Muscle Stretching has no Influence on Knee Joint Proprioception

**DOI:** 10.2478/v10078-012-0061-1

**Published:** 2012-10-23

**Authors:** Rui Torres, José Alberto Duarte, Jan MH Cabri

**Affiliations:** 1 North Polytechnic Institute of Health, Department of Physiotherapy, Paredes, Portugal.; 2 Technical University of Lisbon, Faculty of Human Kinetics, CIPER, Lisbon, Portugal.; 3 University of Porto, Sport Biology, Porto, Portugal.; 4 Norwegian School of Sport Sciences, Department of Physical Performance, Oslo, Norway.

**Keywords:** Intrafusal muscle fibres, extrafusal muscle fibres, sense of joint position, kinaesthesia, force sense

## Abstract

The main objective of this study was to determine if an acute bout of static stretching of the quadriceps muscle affects the sense of joint position, the threshold to detect passive movement, and the sense of force. Thirty young, healthy men (age : 22.1 ± 2.7 years) were randomly divided into two groups. The Stretching Group (n=15) underwent stretching of the dominant quadriceps muscle, which comprised ten passive stretches lasting 30 seconds each, while the Control Group (n=15) remained seated for the same length of time. A repeated-measures analysis of variance was used to establish intragroup differences over time, and an independent sample t-test was used to compare the dependent variables between groups at each moment. None of the measurements revealed any significant change between both groups in each assessment moment or between moments within groups (p>0.05). This study demonstrated that static quadriceps muscle stretching has no effect on the sense of knee joint position, threshold to detect passive movement, and force sense, suggesting that stretching does not have appreciable effect on the spindle firing characteristics and tendon organs activation.

## Introduction

Current literature has questioned the importance of muscle stretching in warm-up exercises before sport practices, particularly those whose success is related to maximal strength or power (Shrier, 2004). Despite this suggestion, passive muscle stretching before or after physical activity is a common practice among athletes and coaches, in the belief that induced stretching flexibility will improve performance (Chan et al., 2001), reduce the risk of injury (Herbert and Gabriel, 2002), and contribute to the recovery of muscle function after intense exercise (Lund et al., 1998).

However, it is known that passive muscle stretching may change the electrical and mechanical properties of the muscle (Avela et al., 1999; Guissard and Duchateau, 2006); slow muscle lengthening of a muscle-tendon unit (contrary to fast muscle stretching) decreases spinal reflex excitability, reducing muscle stiffness and increasing joint range of motion (Avela et al., 1999; Guissard and Duchateau, 2006).

Moreover, it is well known that viscoelastic stress is a mechanic effect induced by passive muscle stretching, which leads to an increase in the compliance of the muscle-tendon unit (Guissard and Duchateau, 2004; Lieber et al., 1991). This increased compliance of the muscle-tendon unit may directly impair its force-generating capacity (Avela et al., 1999; Fowles et al., 2000), and also influence neural activation patterns (Fowles et al., 2000). As a result, acute changes in force production following stretching could apparently affect effort sensation and increase the error in the sense of force.

Independently of the effect of stretching on muscle performance being explained by the changes in neuromuscular transmission and/or biomechanical properties of the muscle, it seems to be clear that changes, which affect the ability to generate force, occur in the activity of the extrafusal muscle fibres.

Consequently, knowing that the stretching effect is not restricted to the common muscle fibres (Guissard and Duchateau, 2004), it is rational to admit that both extrafusal and intrafusal fibres might be involved and impaired. Thus, if the intrafusal muscle fibres suffered parallel alterations, muscle spindles would change their discharge levels and changes in the sensory-motor system might be expected.

Indeed, the importance of the muscle spindles in neuromuscular function is well known, being responsible for conveying information regarding muscle length and rate changes in length (Riemann and Lephart, 2002a). According to this, changes in muscle spindle sensitivity, due to stretching, are expected, leading to alterations in sensory information with an impact on the joint position sense (JPS) and the threshold to detect passive movement (TTDPM) (Bjorklund et al., 2006). Thus, although the sensory-motor system is a complex mechanism involving higher centers of the nervous system to ensure the generation of the correct patterns of muscle activity (Riemann and Lephart, 2002b), it is reasonable to think that changes in the sensory receptors, for example from muscle receptors, should lead to a decrement in the joint proprioceptive acuity.

However, contrasting with the concern of researchers describing the effect of stretching on the extrafusal muscle fibres, the effect of stretching on the indirect markers of muscle spindles and organ tendons functioning is fairly well-studied. For this reason, it becomes necessary to clarify if acute muscle stretching performed prior to physical exercise really compromises joint proprioceptive acuity. In this sense, the present study’s aim was to assess whether an acute bout of static quadriceps muscle stretching affects the knee joint proprioception, specifically in different modalities commonly used in order to assess proprioception sense such as JPS, TTDPM, and the sense of force.

## Material & Methods

### Participants

Thirty healthy, untrained, young males were recruited for this study (age = 22.1 ±2.7 years; body mass = 72.4 ±6.8 kg; body height = 176.6 ±5.3 cm; body mass index = 23.2 ±1.6 kg/m^2^ and maximal isometric peak torque = 220.2 ±31.5 Nm). None of the participants had a history of knee injury and all were free of orthopaedic abnormalities. The participants had not been involved in any stretching and/or a resistance training program for six months prior to the study.

The sample was randomly divided into two groups: the Stretching Group (n=15), which performed 6.5 minutes of stretching and the Control Group (n=15), which remained seated for the same period of time.

### Procedures

The study was performed in accordance with the ethical standards (Harriss and Atkinson, 2009). Moreover, the local Ethics Committee, in accordance with the Helsinki Declaration, approved all procedures prior to the start of this investigation. All volunteers completed a medical screening questionnaire and provided written informed consent prior to participation.

The Stretching Group performed a bout of stretching focusing on their dominant quadriceps muscle, which included ten passive stretches lasting 30 s each with a 10 s rest between stretches (Torres et al., 2007). All passive stretching was observed by the same examiner, who limited the stretch until he felt reasonable resistance or the subject reported discomfort (Johansson et al., 1999). The subject was in a standing position with one knee resting on a chair. The dominant leg was kept relaxed; the examiner passively stretched the quadriceps, flexing maximally the subject’s knee and extending the hip to a neutral position. If maximal knee flexion did not produce the sensation of a stretch or resistance against the movement, hip extension would be added in order to increase the stretch. No intervention was made in the Control Group, which remained seated while the stretching program was conducted.

The dependent variables included knee JPS, TTDPM, and the sense of force, which were recorded in random order before, immediately afterward, and one hour after the stretching program. The protocol for the JPS assessment involved passive positioning and active repositioning (passive-active test) of the dominant leg (Zhou et al., 2008). JPS measurements were performed with an isokinetic dynamometer (Biodex Medical Systems, Inc., Shirley, NY, USA) (Callaghan et al., 2002). The Biodex System 3 isokinetic dynamometer is a mechanically reliable instrument for the measurement of an angular position, isometric torque, and slow to moderately high velocities, with high intra-class correlation coefficients (ICC _2,K_ = 0.99 for each variable) (Drouin et al., 2004). Test instructions were given to the participants prior to their initiation and they were allowed to familiarise themselves with the Biodex System one day before the test.

The participants were seated in the dynamometer chair at 90 degrees of hip flexion with their eyes closed. They were given headphones and were fitted with an air cushion above the leg, which was inflated to a pressure of 40 mmHg to minimize cutaneous sensory information (Callaghan et al., 2002). All participants had the “hold” button in one hand so that they could stop the dynamometer’s lever arm with their thumb when they thought it was at the target angle (Willems et al., 2002). Briefly, for each trial the lower leg was passively moved at 10 degrees per second and positioned at an index angle of either 30 or 70 degrees. The target position was maintained for 5 seconds so participants could memorize the position. Each subject actively reproduced the index angle three times to the best of his ability. The repositioning absolute error was obtained by calculating the mean of the difference (in absolute value) between the target angle and the reproduced angle of the three attempts. The start position was at 100 degrees of the knee flexion and the direction of movement was extension. The order of the tested angles 30 or 70 degrees was randomized. The same researcher always completed the entire JPS assessment protocol and did not provide any feedback to the subject about their performance during the assessment.

The TTDPM in the knee was assessed using the Biodex System (Callaghan et al., 2002). The participants were seated in the same position as described for the JPS evaluation. Additionally, they were blindfolded and had earphones placed over their ears. Each subject was asked to press the handheld stop button when they felt a sensation of movement or a change in the starting knee position, which was engaged at random during the 20s test. Three trials at slow angular velocity (0.25 degrees/s) from starting positions of 30 and 70 degrees knee flexion moving into extension were performed. The average number of degrees in these three consecutive trials was collected to determine the TTDPM in each joint position.

Knee extensor force-matching procedures were conducted at 20% maximal isometric peak torque, which was assessed previous to the study. Isometric sense was utilized to better isolate the role of force (Docherty and Arnold, 2008), and a low load for testing was chosen since the ability to reproduce force is directly related to motor unit recruitment and firing frequency (Cafarelli, 1982). Participants were positioned on the Biodex System in the same manner as for JPS testing and were instructed to obtain the target torque using visual feedback from the dynamometer software. They were then asked to maintain the isometric contraction for 6 s, with a 6 s rest, during six trials. Prior to repeating procedures of the same target torque (but without visual feedback), a 1 min rest period was provided. Participants did not receive feedback about their force-matching performance throughout the test.

The dynamometer software provided the average peak torque over 6 s of the six repetitions used in analysis, without visual feedback to the subject. The difference between the target torque and the average peak torque produced in absolute value was calculated and used for analysis.

The Biodex System was also used to evaluate the MIVC of the quadriceps muscle; this equipment is frequently used in neuromuscular muscle function studies. Briefly, after a warm-up set of four submaximal muscle actions, participants completed three maximal isometric contractions of 6 s each separated by 30 s of rest. Maximal voluntary isometric torque of the quadriceps was assessed at 45 degrees of flexion. Participants received verbal encouragement and the best performance of the three contractions, provided by the dynamometer software (Biodex System 3 Advantage Software, Biodex Medical System, Inc., Shirley, NY), was used to define the target torque for the force sense.

### Analysis

One week prior to the study a test-retest (n=15) with 48 hours between measures for all analyzed variables was performed. The reliability (Intraclass Correlation Coefficient (ICC_2,3_)) of the sense of position and force, and TTDPM was 0.99, 0.98, 0.99, respectively; and the standard error of measurement [SEM=SD(√1−ICC)] (Beckerman et al., 2001) was 0.15 degrees, 0.18 Nm, and 0.03 degrees/s, respectively (p < 0.05).

Moreover, post hoc analysis of statistical power achieved for the number of participants included was performed with G* Power 3.1 software and it was found to be between medium (0.44 and 0.49 to JPS and the sense of force) and large (0.86 and 0.90 to TTDPM) (Faul et al., 2001).

All data was reported as mean ± standard deviation. The distribution of all variables was examined using the Shapiro-Wilk test and no significant difference was found. Independent-sample t-tests were applied to compare the general characteristics of participants and the intergroup comparison of the variables at each moment. A repeated-measures analysis of variance (ANOVA) was used for intragroup comparison at different moments. The level of significance was set at p < 0.05. The SPSS version 18.0 was used for all analyses (SPSS Inc., Chicago, Illinois).

## Results

All 30 participants completed the study. There were no significant differences between the groups in age (p=0.156), body mass (p = 0.755), body height (p = 0.481), body mass index (p = 0.433), or maximal isometric peak torque (p = 0.134).

### Intergroup comparisons

No significant differences (p > 0.05) between the Stretching Group and the Control Group were found in any of the dependent variables studied (Table 1) at each measurement moment (before, immediately afterward, and one hour post stretching).

### Intragroup changes over time

Changes of all dependent variables over time are also presented in Table 1. The absolute error in estimating the accuracy of the JPS showed that static quadriceps stretching did not significantly interfere in the knee JPS of either of the two joint positions studied (p > 0.05). Furthermore, it can also be noted that no significant differences within groups were observed over time (p > 0.05) regarding the effect of the stretching exercise on the TTDPM. Concerning the force sense, the Stretching Group and the Control Group demonstrated similar acuity to reproduce the target torque imposed by the experimental protocol (p > 0.05).

## Discussion

The results showed that the bout of stretching performed in the quadriceps muscle had no effect on knee JPS, TTDPM, or the sense of force, i.e. the assessments made immediately and at one hour afterwards maintained similar values comparable to baseline.

The short-term negative effect on extrafusal fibers leading to a reduction in muscle performance is well documented. However, its effect on intrafusal muscle fibers is not so well studied. Accordingly, knowing the effect of stretching on the sensitivity of muscle spindles, adding to the fact that muscle spindles have a thixotropic property (Proske et al., 1993), it would be an expected interference in the functioning of these muscle receptors by stretching. Consequently, it was the first objective of this study to analyze the impact of stretching on indirect markers of the function of muscle spindles. As a result, our findings demonstrated the effect of an absence of stretching on JPS and TTDPM; it could mean that stretching does not have a considerable effect on the spindle firing characteristics.

These findings, particularly regarding the JPS, agree with others. Indeed, Bjorkland et al. (2006) observed no effect on the sense of shoulder position after a bout of stretching of the agonist and antagonist muscles of the shoulder complex. Furthermore, Larsen et al. (2005) found no differences in the sense of knee position after stretching quadriceps and hamstring muscles. Consequently our results do not confirm the hypothesis that stretching interferes with the viscoelastic properties of the muscle spindles changing its functioning and altering the proprioceptive input.

Despite being used to assess proprioception in healthy persons (Boerboom et al., 2008), TTDPM has not been used to assess the effect of muscle stretching. It has been well documented that TTDPM, as well as JPS, is attributed to the sensitivity of the muscle spindles to detect changes in the muscle length and the velocity of these changes. Therefore, the results of this study showing no changes in the detection of motion after muscle stretching, reinforces the fact that muscle spindles maintain their integrity and normal functioning after a bout of stretching.

Regarding the effect of stretching on tendon organ activation, the sense of force was used as an indirect marker to achieve the other goal of this study, i.e. to determine whether a bout of stretching, which increases the compliance of the muscle-tendon unit, impairs neural activation and leads to an increase in the error of effort sensation. Thus, the findings of this study do not suggest that neural and/or mechanical factors imposed by stretching are sufficient to increase the error of the sense of force.

Well known is the fact that sensory inputs are not restricted to Golgi tendon organs and muscle spindles; peripheral receptors from cutaneous and articular tissues also contribute to afferent proprioceptive inputs. Moreover, the descending commands from supraspinal areas, as a response to the different afferent stimuli, converge collectively onto the static and dynamic gamma motoneurons, leading to a change in the sensitivity of muscle spindles (Riemann and Lephart, 2002b).

However, our opinion is that stretching exercise, as used in this study, might have fundamental effects on muscle receptors, which could mean that sensory information conveyed by other receptors is sufficient to maintain normal levels of proprioception.

Also, a question could arise whether the bout of stretching used in the present study was a significant enough stimulus to induce muscle spindles dysfunction. However, the stretching program chosen for this study consisted of ten passive stretches each lasting 30 s, which can be considered a high volume. Believing that stretching protocols habitually used in sports practices have a lower volume, it would be plausible to defend the statement that stretching generally used before exercising will not affect proprioception.

Although the Biodex is commonly used to assess the sense of joint position and threshold to detect passive motion, we recognize some limitations in this equipment. Particularly the axis of the dynamometer does not accompany the axis of the knee movement, which can lead to a decline in the accuracy of the results.

Due to the fact that static stretching could induce muscle damage in contrast to dynamic stretching (Smith et al., 1993), we chose to analyse only the effect of this type of muscle stretching. Nevertheless, there are other ways of muscle stretching such as dynamic muscle stretching and Proprioceptive Neuromuscular Facilitation Stretching which need further research to examine their effect on joint proprioception.

### Practical implications

Although these findings showed that stretching has no effect on proprioception, it is prudent to keep in mind its negative effect when maximal muscular strength and power are concerned. Thus, this study also contributes to a better understanding of the effect of a stretching during warm-up procedures prior physical activity.

## Conclusion

Acute effect of static muscle stretching has no interference in joint proprioception. Indeed, the bout of stretching used in this study had no influence on all variables assessed, such as the sense of joint position, threshold to detect passive movement, and the sense of force. Consequently, stretching does not suggest having sufficient influence on the muscle receptors functioning, which could compromise joint proprioception.

## Figures and Tables

**Table 1:** Intragroup and intergroup comparison of absolute values of the dependent variables recorded before, immediately afterward, and one hour after stretching.

**Dependent variables**	**Group**	**Before**	**After**	**1 Hour**	**p**
Joint position sense at 30 degrees of knee flexion (degrees)	Control	2.8±1.6	2.5±1.3	2.6±1.3	0.823
	Experimental	2.6±1.5	2.8±1.3	2.3±1.3	0.530
		p=0.754	p=0.643	p=0.477	
Joint position sense at 70 degrees of knee flexion (degrees)	Control	2.9±1.8	2.8±1.3	2.8±1.4	0.982
	Experimental	2.7±1.5	2.5±1.2	3.2±1.9	0.597
		p=0.663	p=0.610	p=0.538	
Threshold to detect passive motion at 30 degrees of knee flexion (degrees)	Control	0.62±0.34	0.64±0.37	0.66±0.22	0.546
	Experimental	0.67±0.28	0.62±0.30	0.70±0.23	0.801
		p=0.665	p=0.549	p=0.648	
				
Threshold to detect passive motion at 70 degrees of knee flexion (degrees)	Control	0.75±0.35	0.73±0.46	0.71±0.35	0.752
	Experimental	0.71±0.17	0.63±0.37	0.66±0.36	0.873
		p=0.945	p=0.352	p=0.621	
Force sense (Nm)	Control	3.9±1.6	3.6±2.0	4.2±2.4	0.775
	Experimental	3.6±2.4	4.1±2.3	3.9±2.7	0.686
		p=0.554	p=0.450	p=0.961	
Maximal isometric voluntary contraction (Nm)	Control	222.2±23.9	226, 1±27.9	216.9±30.4	0.634
	Experimental	215.5±24.9	218.8±32.1	220.3±28.1	0.455
		p=0.880	p=0.418	p=0.983	

Values are expressed as mean ± standard deviation (p<0.05).
